# Detection of Mesenchymal Stem Cell Aging Using an Integrin Mechano‐Probe

**DOI:** 10.1111/nyas.70247

**Published:** 2026-04-09

**Authors:** Aimei Liu, Xiaojun Liu, Juan Li, Jiangtao Li, Xinpeng Wang, Yuanjun Dong, Feng Shao, Mingjun Bi, Xiaoyan Deng, Guixue Wang, Yongliang Wang

**Affiliations:** ^1^ School of Life Sciences and Health University of Health and Rehabilitation Sciences Qingdao Shandong PR China; ^2^ Qingdao Municipal Hospital University of Health and Rehabilitation Sciences Qingdao Shandong PR China; ^3^ Qingdao Central Hospital University of Health and Rehabilitation Sciences Qingdao PR China; ^4^ Department of Quality Management Qingdao Special Servicemen Recuperation Center of PLA NAVY Qingdao PR China; ^5^ School of Rehabilitation Science and Engineering University of Health and Rehabilitation Sciences Qingdao Shandong PR China; ^6^ School of Social Development University of Health and Rehabilitation Sciences Qingdao Shandong PR China; ^7^ Key Laboratory of Marine Chemistry Theory and Technology, Ministry of Education, College of Chemistry and Chemical Engineering Ocean University of China Qingdao PR China; ^8^ Advanced Medical Research Institute, Cheeloo, College of Medicine Shandong University Jinan PR China; ^9^ Key Laboratory of Biomechanics and Mechanobiology of Ministry of Education, Beijing Advanced Innovation Center For Biomedical Engineering, School of Biological Science and Medical Engineering Beihang University Beijing PR China; ^10^ Key Laboratory for Biorheological Science and Technology of Ministry of Education, State and Local Joint Engineering Laboratory For Vascular Implants Bioengineering College of Chongqing University Chongqing PR China; ^11^ Institute of Panvascular Biology JinFeng Laboratory Chongqing PR China; ^12^ School of Economics and Management Qingdao Institute of Technology Qingdao PR China

**Keywords:** aging, GFP probe, integrin tension, mesenchymal stem cell

## Abstract

Mesenchymal stem cells (MSCs) play a crucial role in cell therapy, but their efficacy diminishes with age. While biochemical methods like flow cytometry, immunoblotting, β‐galactosidase labeling, and gene profiling are effective for screening MSC aging, mechanical factors also change with aging. After analyzing the transcriptomes of young and aged MSCs, we observed downregulation of adhesion‐related genes and, based on those data, developed an integrin mechano‐probe to measure integrin forces in MSCs. The probe's effectiveness was validated by mapping integrin forces in young and aged MSCs, revealing reduced integrin force signals in aged cells that correlated with downregulated expression of β1 integrins. This finding was further confirmed by flow cytometry and β‐galactosidase staining. Overexpression of integrin β1 in aged MSCs restored mechanical signaling and increased p‐ERK production, suggesting that the integrin β1/ERK pathway plays a role in rescuing the contractility of aged MSCs. Our mechano‐probe enables distinguishing aged MSCs in a mixed pool of young and aged MSCs and identifying aged MSCs taken from mouse tissue. Integrin tension signals, as measured by a mechano‐probe, may thus complement biochemical markers in assessing MSC aging.

## 1 Introduction

Mesenchymal stem cells (MSCs) are stromal cells that can differentiate into chondrocytes, myocytes, adipocytes, and osteocytes. This multipotent nature renders MSCs highly valuable in the field of tissue regeneration, offering promising avenues for therapeutic interventions. The application of MSC‐based cell therapy has demonstrated considerable efficacy in addressing cardiovascular dysfunction and nerve diseases [1, 2], underscoring its potential as a therapeutic modality in clinical settings. MSC differentiation is sensitive to microenvironmental factors such as cytokines and mechanical changes. Moreover, aging poses significant challenges to MSC functionality, as evidenced by reduced responsiveness to microenvironmental signals. This age‐related decline is often characterized by diminished proliferation rates and an increase in cell volume, highlighting the dynamic interplay between MSCs and their local signaling milieu [3]. Consequently, characterizing the aging status of MSCs is crucial for achieving better results in patient treatment with MSCs or extracellular vesicles derived from MSCs.

Aging is a complex and continuous process that gradually impacts the human body. At the cellular level, aging often leads to a reduction in cell proliferation, diminishing the regenerative capacity of tissues. For instance, aging of dermal fibroblasts results in reduced deposition of the extracellular matrix (ECM), altering tissue structure and diminishing skin elasticity [4]. Similarly, aging affects bone composition, leading to a more brittle bone architecture [5]. In certain age‐related pathological conditions, arterial elasticity and residual wall stress decrease, increasing the risk of cardiovascular disease among the elderly [6]. This is attributed to the loss of vascular cells’ ability to adapt to external stressors. Additionally, aging is associated with dysfunction in the muscle system, including a decrease in skeletal muscle strength alongside increased stiffness and inelasticity [7]. Moreover, aging contributes to impaired immunity, characterized by delayed immune cell migration and a lack of effective communication between T cells and antigen‐presenting cells (APCs) [8]. These factors collectively contribute to compromised immune function in aging individuals.

Integrins are transmembrane receptors that play a crucial role in cell adhesion and signaling, making them key players in cell mechanics. These heterodimeric proteins consist of α and β subunits and bind to ECM proteins, such as fibronectin, collagen, and laminin, as well as cell surface ligands like ICAM‐1 and VCAM‐1 [9, 10]. One of the fundamental functions of integrins is their involvement in cell adhesion and mechanotransduction. Integrins anchor cells to the ECM and transmit mechanical forces between the extracellular environment and the intracellular cytoskeleton. Integrin‐mediated mechanotransduction pathways, including Rho GTPases, focal adhesion kinase (FAK), and Yes‐associated protein (YAP)/transcriptional coactivator with PDZ‐binding motif (TAZ), play crucial roles in translating mechanical cues into biochemical signals that regulate gene expression, cell proliferation, and differentiation [11, 12, 13]. With aging, alterations in integrin expression and activity can impact MSC–ECM interactions, leading to changes in cell adhesion strength, cytoskeletal organization, and migratory capacity. Integrin‐mediated activation of downstream pathways, such as PI3K/AKT, MAPK/ERK, and Wnt/β‐catenin, regulates cellular processes involved in aging [14, 15], such as oxidative stress response, DNA damage repair, and stem cell renewal [16, 17]. Furthermore, reduced expression of integrin β1 serves as a marker for human skin aging [18], which could be valuable for mechanical characterization during the aging process.

Researchers have endeavored to identify biomarkers for aging by analyzing biochemical and biophysical properties. In various cell types, such as vascular smooth muscle cells, myocytes, cardiac fibroblasts, and cardiomyocytes, tension force tends to increase with aging [19]. However, skin fibroblasts and osteocytes exhibit the opposite trend [19, 20]. A common parameter among these cells and others, including macrophages, lymphocytes, red blood cells, and trabecular meshwork cells, is the augmentation of stiffness during aging [21, 22, 23, 24]. In MSCs, several key biomarkers of aging have been identified, including upregulation of p53 and p21 [25], DNA damage, expression of senescence‐associated β‐galactosidase (SA‐β‐gal) [26], cell‐cycle arrest, morphological changes [27], altered differentiation patterns, compromised colony‐forming ability, and induction of a senescence‐associated secretory phenotype (SASP) [28]. Two reports have demonstrated mechanical analysis for MSCs [29, 30]. While mechanobiological changes have been studied extensively in other cell types, reports specifically addressing the mechano‐related changes in aging MSCs, particularly regarding integrin force transmission, are limited.

Our study focuses on evaluating the mechanical properties of MSCs, particularly in terms of integrin tension. We employed a GFP‐RGD mechano‐probe, including anchoring the probe on glass‐bottom Petri dishes using biotin–avidin, to measure the pulling forces exerted on integrins in MSCs. GFP has been observed to unfold at approximately 60 pN at a pulling speed of 100 nm/s [31], and the adhesion forces between individual biotin–avidin pairs have been measured at 160 pN [32]. Our findings show a decline in integrin tension with aging by use of a mechano‐probe for assessing MSCs in the context of aging, offering insights for future evaluations. Our mechanistic study indicated that the downregulation of integrin β1 may explain the reduced contractility of aged MSCs. However, further research is needed to elucidate the mechanism responsible for lower contraction generated by MSCs through integrins.

## 2 Materials and Methods

### 2.1 Mechano‐Probe Design

The force sensor was created using green fluorescent protein (GFP) and an RGD motif from fibronectin, which was linked to an Avi‐tag at the N‐terminal for biotin incorporation. The plasmids containing this construct and BirA were coexpressed in *E. coli* strain BL21 and the force sensor protein tagged with biotin was subsequently purified using affinity chromatography. Below is the detailed amino acid sequence of the sensor:

**Avi**‐*GFP*‐RGD‐8xHis:

MGSS**GLNDIFEAQKIEWHE**SEFG*SSKGEELFTGVVPILVELDGDVNGHKFSVRGEGEGDATIGKLTLKFICTTGKLPVPWPTLVTTLTYGVQCFSRYPDHMKRHDFFKSAMPEGYVQERTISFKDDGKYKTRAVVKFEGDTLVNRIELKGTDFKEDGNILGHKLEYNFNSHNVYITADKQKNGIKANFTVRHNVEDGSVQLADHYQQNTPIGDGPVLLPDNHYLSTQTVLSKDPNEKGSENLYFQGIEGRHSGSGSRDHMVLHEYVNAAGIT*KLAATVYAVTGRGDSPASSKLAAHHHHHHHH

### 2.2 MSC Culture

Human MSCs were purchased from Guangzhou Saliai Stem Cell Science and Technology Co. LTD (Guangzhou, China). The cells were cultured in DMEM/F12 medium supplemented with 10% fetal bovine serum (FBS, 10099‐141, Gibco) and 1% penicillin–streptomycin (15140122, Gibco). To obtain aged MSCs, a passaging method was employed, and MSCs at different passages (e.g., P5, P8, P15) were utilized for integrin force analysis. To estimate the effects of integrin β1 (ITG‐β1) in aged MSCs, lentivirus (2 × 10^9^ TU/mL) encoding ITG‐β1, LV‐ITG‐β1, was added to the culture medium and then fresh culture medium was added 24 h later. The expression of ITG‐β1 was detected by immunoblots.

### 2.3 Isolation and Culture of Adipose‐Derived Mesenchymal Stem Cells

Animal procedures were approved by the Laboratory Animal Ethics Committee of the University of Health and Rehabilitation Sciences, China (approval No. 20232012). C57BL/6 mice were maintained in a specific‐pathogen‐free facility (25°C, 70% relative humidity, 12 h light/12 h dark cycle) with ad libitum access to autoclaved water and irradiated chow. Mice were euthanized by cervical dislocation. In a sterile field, inguinal fat pads were excised and washed three times with ice‐cold PBS containing 1% penicillin–streptomycin (P/S). The tissue was minced into <1 mm^3^ fragments and digested in 0.1% Type‐I collagenase (2–3 volumes relative to tissue) for 60 min at 37°C with gentle agitation. Enzyme was inactivated by adding PBS to 50 mL, and then the suspension was filtered through a 100 µm cell strainer and centrifuged at 300 × *g* for 5 min. The pellet was resuspended in DMEM/F12 medium supplemented with 10% FBS and 1% P/S and then incubated at 37°C with 5% CO_2_. After 3 h, nonadherent cells were washed away by a medium change; adherent cells were adipose‐derived mesenchymal stem cells (AMSCs).

### 2.4 Preparation of Mechano‐Probe Surfaces

All probes were immobilized on a glass surface through NeutrAvidin–biotin affinity. Glass bottom Petri dish (FCFC016, BeyoGold35 mm confocal dishes, Φ14 mm glass) was incubated with 200 µL 500 µg/mL bovine serum albumin (BSA)–biotin (BSA, A8549, Sigma‐Aldrich) in PBS for 20 min, and then the surface was washed by PBS solution three times. BSA–biotin provides biotin tags for NeutrAvidin coating and suppresses nonspecific cell adhesion.

The surface was then incubated with 200 µL 100 µg/mL NeutrAvidin (31000, Thermo Fisher Scientific Inc.) for 30 min and then the surface was washed with PBS solution three times. A total of 200 µL 1 mg/mL biotin–GFP–RGD was then incubated on the NeutrAvidin surface for 30 min, after which the surface was washed by PBS three times without drying.

### 2.5 Fluorescence Photography and Fluorescence Loss Analysis

Prepared samples were imaged using a Nikon ECLIPSE Ti2 microscope. Fluorescence loss was observed and was correlated with cell plating location. The optical filters used for GFP fluorescence loss were a 480/30 nm (center wavelength/bandwidth) excitation filter, a dichroic mirror with a 505 nm cutoff, and a 512–558 nm emission filter.

The relative average fluorescence loss was evaluated via the cross‐sectional analysis of the fluorescence intensity near focal adhesion regions or the cell edges using a custom‐written MATLAB code. The relative average fluorescence loss is defined as (*I*_background_ − *I*_loss_)/*I*_background_, where *I*_background_ is the grayscale fluorescence intensity outside the focal adhesion region, and *I*_loss_ is the grayscale fluorescence intensity of the focal adhesion region that shows fluorescence loss.

### 2.6 Flow Cytometry

Flow cytometry was used to monitor the cell senescence using the CellEvent Senescence Green Flow Cytometry Assay Kit (C10840, Invitrogen). Briefly, 1 × 10^6^ cells were collected, fixed for 10 min, stained for 1 h at 37°C without CO_2_, and evaluated by flow cytometry.

### 2.7 Beta‐Galactosidase Staining

MSCs were stained with β‐galactosidase using the Senescence β‐Galactosidase Staining Kit (C0602, Beyotime, Shanghai, China) following the manufacturer's instructions. Briefly, MSCs from the dish were washed with PBS and then 1 mL of β‐galactosidase stain fixing solution was added at room temperature for 15 min. After washing the MSCs three times with PBS, 1 mL of dye solution was added and the cells were incubated overnight at 37°C [this step cannot be conducted in carbon dioxide incubators]. The stained cells were then viewed and photographed under an ordinary light microscope.

### 2.8 Immunoblot Analysis

Proteins extracted from cultured MSCs were separated on 10% sodium dodecyl sulfate polyacrylamide gels and subsequently transferred to 0.45 µm polyvinylidene fluoride membranes. Primary antibodies used included integrin β1 rabbit monoclonal antibody (R24729, Zenbio, Chengdu, China) at a dilution of 1:1000, GAPDH rabbit monoclonal antibody (AF1186, Beyotime,) at a dilution of 1:1000, p53(2D10) rabbit monoclonal antibody (Cat. No. 250143, Zenbio, Chengdu, China), p21 rabbit monoclonal antibody (R25235, Zenbio, Chengdu, China), ERK1/2 (343830, Zenbio), p‐ERK1/2 (R22900, Zenbio); these antibodies were incubated overnight at 4°C. The secondary antibody used was peroxidase anti‐rabbit IgG (A0208, Beyotime, Shanghai, China) at a dilution of 1:5000, incubated for 1 h at room temperature. Signal detection was performed using a luminescence kit (Cat# WBKLS0100; Millipore, Bedford, MA, USA) and the intensity of bands was quantified using ImageJ software (National Institutes of Health, Bethesda, MD, USA). All experiments were conducted in triplicate.

### 2.9 Immunostaining

The cells were fixed with 4% (w/v) formaldehyde for 15 min at room temperature and then rinsed three times with PBS. Subsequently, cells were permeabilized with 0.5% Triton X100 detergent at room temperature for 10 min and washed three times with PBS. Following cell fixation and permeabilization, 3% BSA was added to the cell samples and incubated at room temperature for 1 h. The cells were then rinsed three times with PBS. Then, anti‐vinculin antibody (Lot 2431483, Invitrogen) was added at a 1:200 dilution and incubated for 1 h at room temperature. Subsequently, samples were rinsed three times with PBS containing 0.05% Tween 20, incubated for 5 min each time. Imaging was performed using a 40× objective lens on a Nikon ECLIPSE Ti2 fluorescence microscope.

### 2.10 RNA Sequencing

MSC were collected for RNA sequencing (RNAseq), which was performed by the Majorbio Company (Shanghai, China). Briefly, RNA was extracted from MSC using the TRIzol (15596026CN, Invitrogen) and mRNA was enriched using Oligo(dT) magnetic beads. cDNA was synthesized using reverse transcriptase after mRNA fragmentation, and then PCR was performed to obtain the final library. The expression of gene transcripts (RNA‐Seq data) were quantitatively analyzed by RSEM, and differentially expressed genes (DEGs) were identified using the DESeq2 R package (1.26.0). Genes with FDR <  0.05 and |fold change| > 1.5 were set as the threshold. The Goatools and Python SciPy package was used for GO enrichment and KEGG analysis. Three independent samples were used in each group.

### 2.11 Statistical Analysis

All data were presented as mean ± SEM. GraphPad Prism 8.0 software was used for statistical analyses. Data were analyzed using Student's *t*‐test for two groups and the differences in means among multiple groups were assessed using one‐way ANOVA with Tukey's multiple comparison. A *p* value of less than 0.05 was considered indicative of a significant difference.

## 3 Results and Discussion

### 3.1 Altered Transcriptomic Profiles and Adhesion‐Related Signaling in Aged MSCs

**FIGURE 1:**
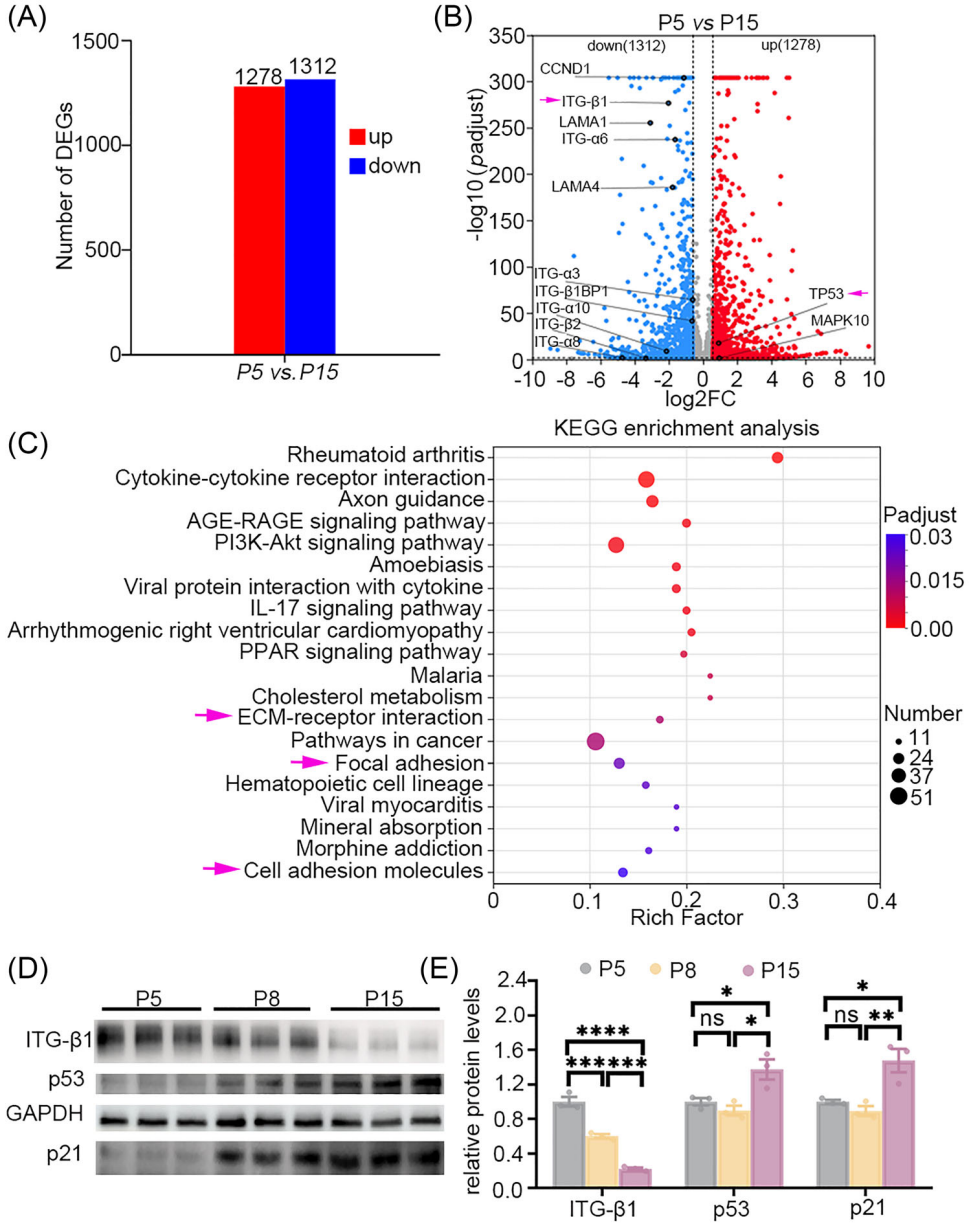
Altered transcriptomic profiles in aged MSCs. (A) RNAseq of P5 and P15 MSCs revealed 1278 upregulated DEGs and 1312 downregulated DEGs in the P15 MSCs compared to the P5 MSCs (FDR < 0.05, |Fold Change| > 1.5). (B) Volcano diagram showing up‐ and downregulated genes in MSCs. (C) DEGs clustering in ECM–receptor interaction, focal adhesion, and cell adhesion molecules signaling pathway using KEGG pathway enrichment analysis. (D, E) Immunoblot showing expression of ITG‐β1, p53, and p21 in P5, P8, and P15 MSCs (D), with a significant downregulation of ITG‐β1 and a significant upregulation of p53 and p21 in P15 MSCs compared with that in the P5 and P8 MSCs (E). Data are presented as mean ± SEM. *p < 0.05, **p < 0.01, ***p < 0.001, ****p < 0.0001, *n* = 3, one‐way ANOVA with Tukey's multiple comparison in E.

Cell aging is a continuous process that requires extensive investigation, and identifying markers for MSC senescence is essential. Profiling mRNA and protein expression at different MSC passages can provide valuable insights for biomarker discovery. To explore the molecular differences between young (P5, primary cell fifth generation) and aged (P15, primary cell 15th generation) MSCs, we performed RNAseq on RNA extracts from P5 and P15 MSCs. We identified 2590 DEGs (FDR <0.05, |Fold Change| >1.5) between the P5 and P15 groups, including 1278 DEGs with upregulation and 1312 DEGs with downregulation (Figure 1A). The downregulation of adhesion‐related genes, such as ITG‐β1, ITG‐β2, ITG‐α3, ITG‐α6, LAMA1, and LAMA4, were identified in the volcano diagram of 1312 DEGs (Figure 1B). Meanwhile, the TP53 gene is upregulated in the volcano diagram of 1278 DEGs. We performed KEGG pathway enrichment analysis of 2590 DEGs and found that these DEGs were clustered in signaling pathways related to ECM–receptor interaction, focal adhesion, and cell adhesion molecules (Figure 1C). Based on these findings, we speculate that the adhesion‐related functions of MSC cells may change with aging.

Immunoblot analysis of aged MSC extracts confirmed significant downregulation of ITG‐β1 and upregulation of p53 and p21 compared to young MSCs (Figures 1D,E), results further validated by qPCR (Figure S1). Numerous studies underscore the critical role of p53 in maintaining genomic integrity, particularly in the DNA damage response. In senescent cells, p53β is upregulated through SRSF3‐mediated splicing [33]. Acetylation of p53 at specific sites inhibits the phosphorylation of certain serines in the N‐terminal region, thereby facilitating the activation of p53‐target genes such as CDKN1A, which encodes p21^Cip1/Waf1^, a crucial regulator in the senescence pathway [34]. Additionally, aging is linked to a decrease in smooth muscle α‐actin stress fibers and changes in protein recruitment to cell‐matrix adhesions [14]. These mechanical changes suggest that single‐cell mechanics could serve as a marker for identifying senescent cells. Therefore, the force profile generated by integrin β1 may serve as a reliable method for detecting aging MSCs, which prompted our design of a probe to monitor integrin tension in MSCs.

Although the mRNA levels of p53 and p21 were upregulated in P8 cells compared with P5 cells (Figure S1), no corresponding increase in protein expression was observed. This discrepancy suggests the involvement of more complex regulatory mechanisms, such as post‐transcriptional or epigenetic regulation. Nevertheless, a decline in proliferative capacity was confirmed by the CCK‐8 assay (Figure S2), demonstrating functional aging of the MSCs used in this study. Furthermore, the progressive aging phenotype from P5 to P8 to P15 cells was validated by β‐gal staining (Figure 3).

### 3.2 The Principle of the GFP‐RGD Mechano‐Probe

**FIGURE 2:**
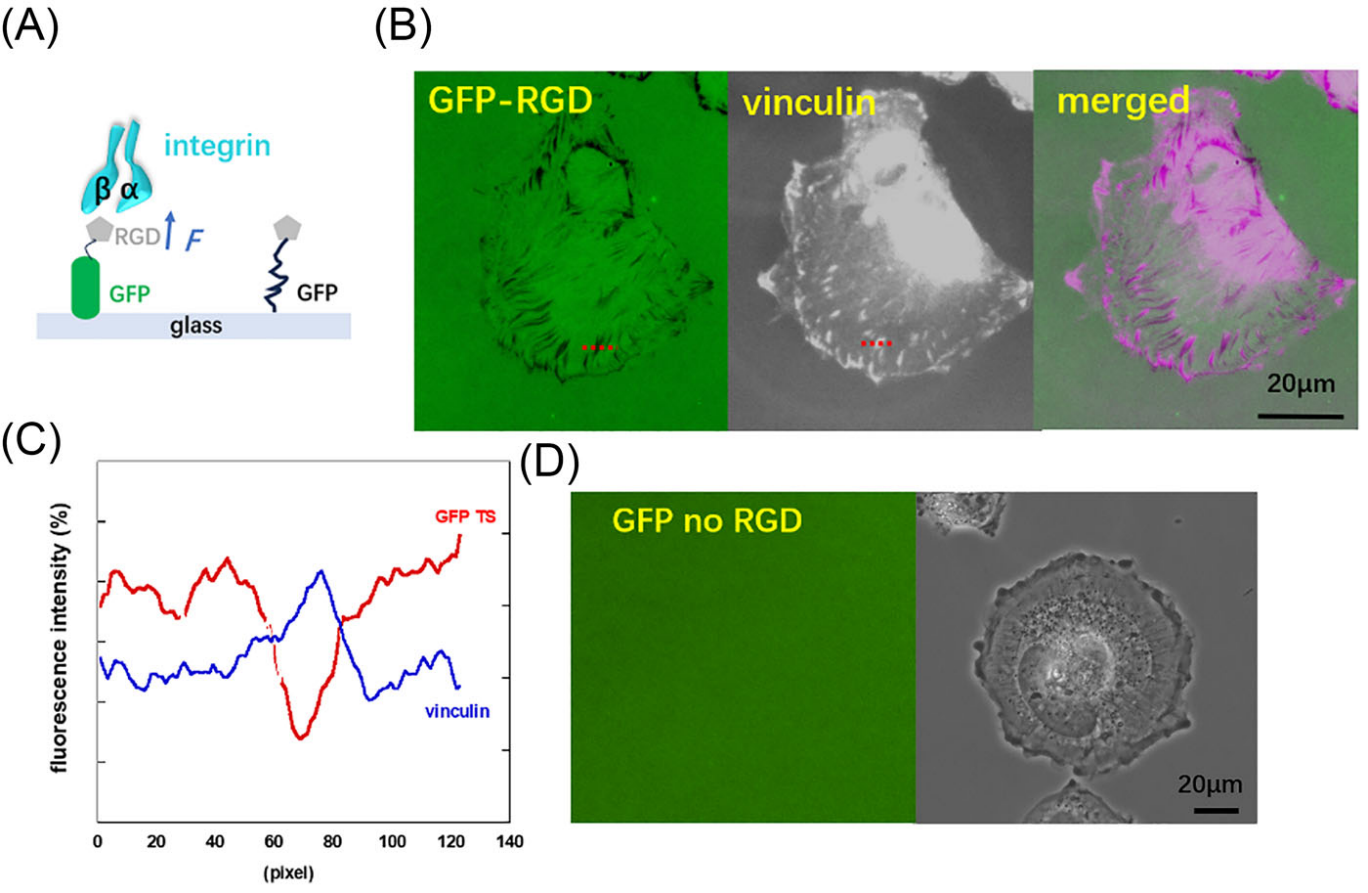
The scheme of the GFP‐RGD mechano‐probe. (A) GFP loses fluorescence when it is stretched by an integrin with enough force. (B) Immunostaining for vinculin demonstrates the focal adhesions were colocalized with force signals. The colocalization of vinculin and tension sensor signals is shown in (C) for the region marked by the red dotted line in (B). (D) Confirmation that GFP alone does not produce a force signal, and thus the signal was not derived from protease degradation.

We had developed a sensor utilizing yellow fluorescence protein (YFP), which has been engineered with a short peptide RGD motif recognized by integrin α5β1, αvβ3, and other integrins [35]. Using this sensor, we monitored the integrin tension in many cell types [36]; multiplexed with ITS, the YFP‐RGD probes revealed that the distinct high‐level integrin tension in cells of different motility [37]. Previously, GFP was calibrated and utilized as a force sensor in the 30‐ to 60‐pN range [38]. Briefly, cells apply traction forces through integrins bound to their ligands (e.g., RGD‐containing peptides); these forces propagate along the mechano‐probe, stressing the GFP module before reaching the avidin–biotin linkage to the substrate. Because the unfolding force of GFP is lower than both the avidin–biotin bond strength and the BSA–glass interaction (typically nN), GFP represents the weakest point in the construct. Thus, GFP unfolding serves as a reporter of the integrin‐generated force threshold. In our study here, we posited that the unfolding of GFP leads to fluorescence loss (see Figure 2A). Figure 2B,C shows that focal adhesions colocalized with the force sensor fluorescence loss.

Focal adhesions consist of several hundreds of proteins, including integrin, vinculin, talin, nuclease and proteinases, etc. [39, 40]. Our previous study detected the surface nuclease‐DNase X on the cell membrane [41]. Since proteases may exist at focal adhesions in MSCs and thus possibly degrade substrates such as GFP, this may cause false positive signals. To rule out this concern, a truncated GFP alone was immobilized on the glass surface; in this case, we observed no fluorescence signal resembling focal adhesions (Figure 2D). Indeed, we found the dsDNA‐based tension sensors produced false positive signals due to MSC degradation, even in the absence of an RGD‐binding motif (Figure S3), demonstrating that the peptide‐based mechano‐probe is particularly suitable for studying the mechanical properties of MSCs.

### 3.3 The Mechano‐Probe Records Integrin Tension in MSCs during Aging

**FIGURE 3:**
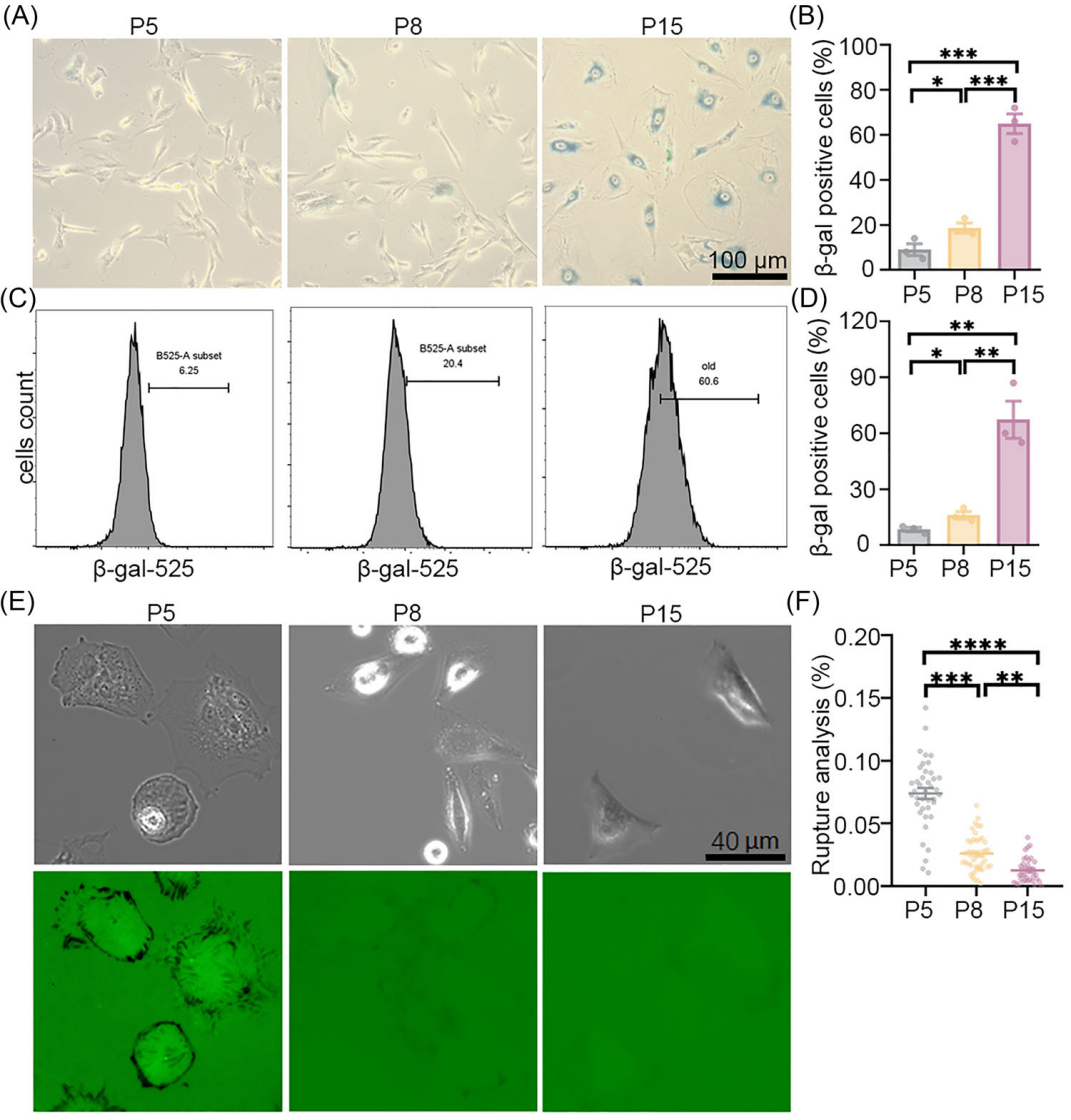
Integrin tension evaluated in young and old MSCs. (A, B) β‐Gal staining for MSCs (A) and the corresponding statistical analysis (B), demonstrating the aging status of the cells. (C, D) The proportion of senescent MSCs measured by flow cytometry (C) and the corresponding statistical analysis (D). (E, F) Force map of MSCs on the tension sensor surface (E) and the corresponding statistical analysis (F). Data are represented as mean ± SEM. *p < 0.05, **p < 0.01, ***p < 0.001, ****p < 0.0001, *n* = 3 in B and D, *n* = 40, 43, and 46 in F. One‐way ANOVA with Tukey's multiple comparison.

MSCs representing various passages from young to aged stages were plated on the sensor surface. The aging status was confirmed by β‐galactosidase staining (Figures 3A,B) and flow cytometry (Figures 3C,D), as described in the Methods. The mechanical signal generated from the unfolding of GFP and/or the dissociation of avidin–biotin complexes was recorded (Figure 2C). Our analysis found a decline in rupture intensity with aging (Figure 3E). Interestingly, MSCs treated with H_2_O_2_ (50 nM, 48 h) exhibited senescence phenotypes but did not show detectable changes in probe response (Figure S4). This suggests that the sensor preferentially detects mechanically relevant changes associated with natural, passage‐induced aging rather than chemically induced senescence.

To further investigate this discrepancy, 3T3L1 cells were treated with doxorubicin (DOX, 200 µM for 1 h), followed by 2 days of recovery in fresh medium prior to β‐gal staining. In parallel, 3T3L1 cells were treated with H_2_O_2_ (50 nM, 48 h) before β‐gal staining. A pronounced reduction in mechanical signal was observed in DOX‐treated cells, whereas no significant change was detected in H_2_O_2_‐treated cells (Figure S5). The mechanistic basis for this difference remains unclear but may reflect distinct pathways underlying replicative‐, genotoxic‐, and oxidative stress–induced senescence. The probe is capable of detecting mechanical alterations in certain drug‐induced senescence models, suggesting its potential utility for drug screening applications. However, its performance appears to be context‐dependent. Further systematic studies comparing the same cell type treated with various senescence inducers are needed to fully validate its broad applicability.

Our findings align with reports showing that aging cells exhibit reduced contractility. Specifically, aged hearts demonstrate decreased contractility at baseline physiological states, highlighting the need for distinct therapeutic approaches to treat cardiac dysfunction in older patients compared to younger ones [42]. In addition, the mechano‐probe enabled analysis of live cells, allowing subsequent reuse of the same cell population, in contrast to β‐gal staining, which requires cell fixation. Moreover, single‐cell analysis using the probe provided insight into cellular heterogeneity, information that cannot be captured by bulk assays such as immunoblot or PCR.

We chose GFP‐RGD as an age mechano‐probe sensor due to limitations encountered with dsDNA‐based sensors (Figure S4), which are prone to degradation at adhesomes [41]. The mechano‐probe sensor construct is not covalently conjugated to the glass surface but has a biotin tag for easier sensor system preparation, unlike covalent immobilization requiring chemical reactions and more time. Nuclease‐resistant tension sensors using short DNA fragments have been designed with peptide nucleotide acid‐DNA pairs [43] or phosphorothioate‐modified DNA [44], but they are more expensive than the mechano‐probe sensor we used. Note that we used protein engineering to overcome nuclease interference, while losing some sensitivity compared to the integrative tension sensor (ITS) [45] or hairpin DNA tension sensors [46]. Recent advancements in DNA‐based tension sensors have broadened our understanding of integrin dynamics in cell adhesion and contraction by allowing the analysis of integrin loading rates [47, 48]. As a result, the MSC mechano‐probe we used could be further refined to capture integrin loading rate differences between aged and young MSCs.

### 3.4 Screening Old MSCs from a Mixed Pool by Using the Mechano‐Probe

**FIGURE 4:**
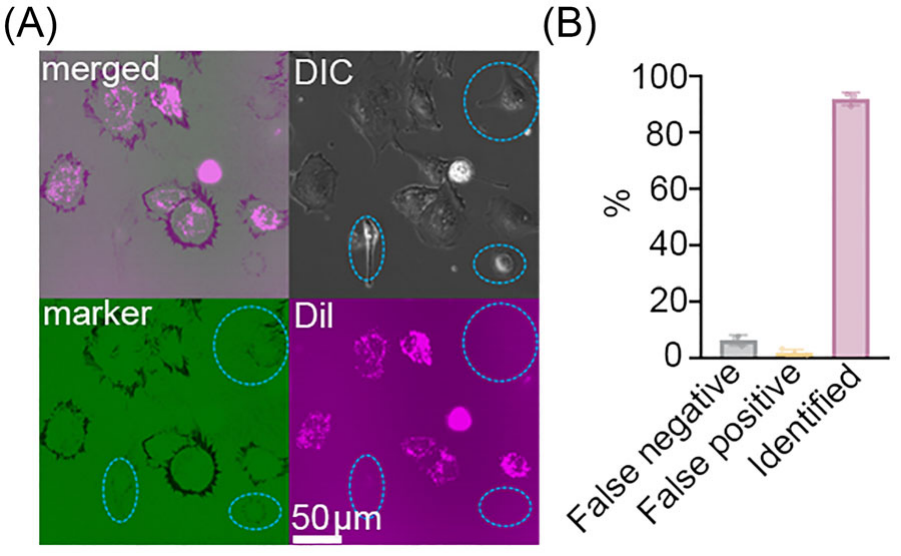
Distinguishing aged and young MSCs in a mixed pool. (A) Aged MSCs mixed with young MSCs labeled with Dil dye were coseeded on the sensor surface for 2 h. The aged cells could be identified by a relatively low mechano‐probe signal; *n* = 100. (B) Calculated efficacy of this method in screening, where the false negative rate is small, approximately 5.4% in total. Note that this evaluation is based on single cells. With multiple MSC cells, the false negative rate would be 0.054*^N^* with *N* as the number of MSC cells (0.054*^N^* is the probability of that none of the *N* cells shows integrin force signal).

To further confirm the efficacy of this method to identify aged MSCs, we prepared an artificial pool of aged and young MSCs with a ratio of 1:5 and plated them on probe‐coated glass surface for 2 h, where the young MSCs were labeled with Dil cell membrane staining. Figure 4 shows that the labeled young MSCs had a higher mechano‐probe signal compared to aged MSCs. Since the adhesion of aged MSCs decreased significantly compared to young MSCs, we reported only the adhered cells. The false negative (young cells showed weaker force signal) rate was 5.4% and the efficacy was around 92.6%, which is acceptable in reporting the cell aging information, as the β‐gal staining method may also generate false positives due to individual manipulation. Aged MSCs could be identified using this simple approach, which would also facilitate the reuse of plated cells for future analyses, such as single‐cell omics.

### 3.5 Overexpressing ITG‐β1 in Aged MSCs Rescues Cell Contractility through the Integrin β1/ERK Pathway

**FIGURE 5:**
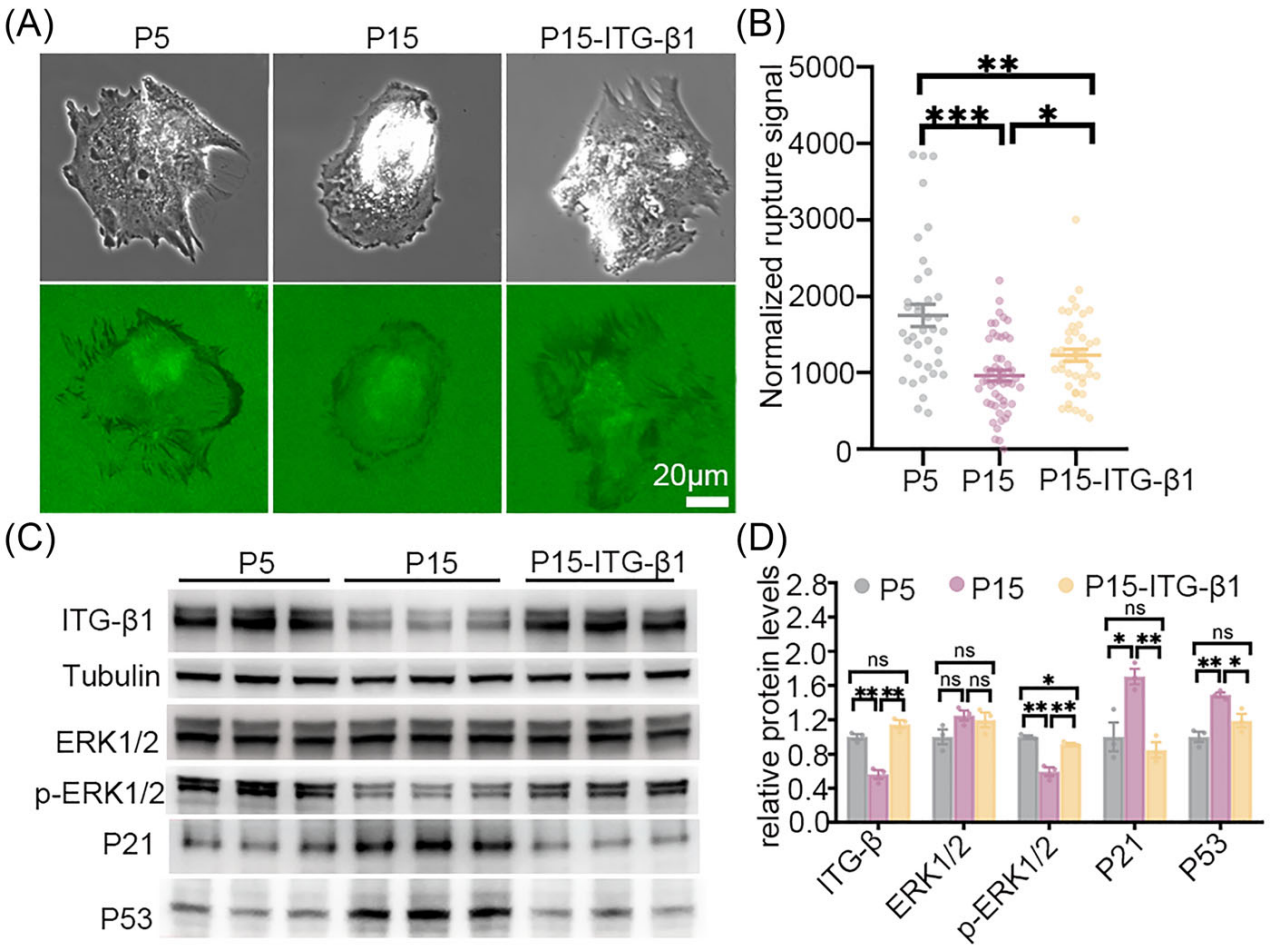
Aged MSCs showed increased force signal after upregulation of integrin β1. (A) A rescue experiment comparing the mechano‐probe signal in three groups: P5, P15, and P15‐ITG‐β1 MSCs. (B) Normalized rupture signal varies, with significant differences between the aged, aged‐ITG β1, and young MSCs. (C, D) Immunoblot analysis revealed the expression of ITG‐β1, ERK1/2, p‐ERK1/2, p21, and p53 in P5, P15, and P15‐ITG‐β1 MSCs. Compared to P5 MSCs, P15 MSCs showed significant downregulation of ITG‐β1 and p‐ERK1/2, along with upregulation of p53 and p21. These changes were reversed upon ITG‐β1 overexpression in aging MSCs, returning to levels close to those in P5 MSCs. Data are represented as mean ± SEM. *p < 0.05, **p < 0.01, ***p < 0.001, *n* = 45 in B and *n* = 3 in D, one‐way ANOVA with Tukey's multiple comparison.

In order to confirm that integrin β1 contributes to the contraction signal in MSCs, we overexpressed integrin β1 (ITG‐β1 OE) in aged MSCs (Figure 5C) and then replated cells of different ages on mechano‐probe surfaces. The mechano‐probes showed that ITG‐β1 OE aged cells had an increased force signal compared to aged cells (1231 ± 521 vs. 965 ± 503, increased by 27.5%), but the intensity was still lower than that in young cells (1231 ± 521 vs. 1698 ± 863; Figures 5A,B). We also observed a significant decrease in p‐ERK expression and marked upregulation of p21 and p53 in aging MSCs (Figures 5C,D). We found upregulation of p‐ERK and downregulation of p21 and p53 after aging MSCs were transfected with the plasmid overexpressing ITG‐β1 (Figures 5C,D). Our result demonstrated that the ITG‐β1 plays a role in cell force generation, especially in the detection of cell aging. Note that integrins are the linker between the ECM and cell cytoskeleton; their expression levels should effectively influence cell adhesion and migration. The successful clustering of integrins propagates signals through a variety of kinases, including FAK, ERK, PI3K, which have a profound influence in cell physiology. As is well known, the ERK and PI3K signaling pathways play crucial roles in cell proliferation and apoptosis resistance [16, 49]. The upregulation of ITG‐β1 can also influence cell growth. Direct pulling force can also be transmitted through nesprin and lamin A [50], influencing the nuclear chromosome packaging and thus gene expression.

### 3.6 The Mechano‐Probe Can Screen MSCs Derived from Young and Old Mice Based on Integrin Tension

**FIGURE 6:**
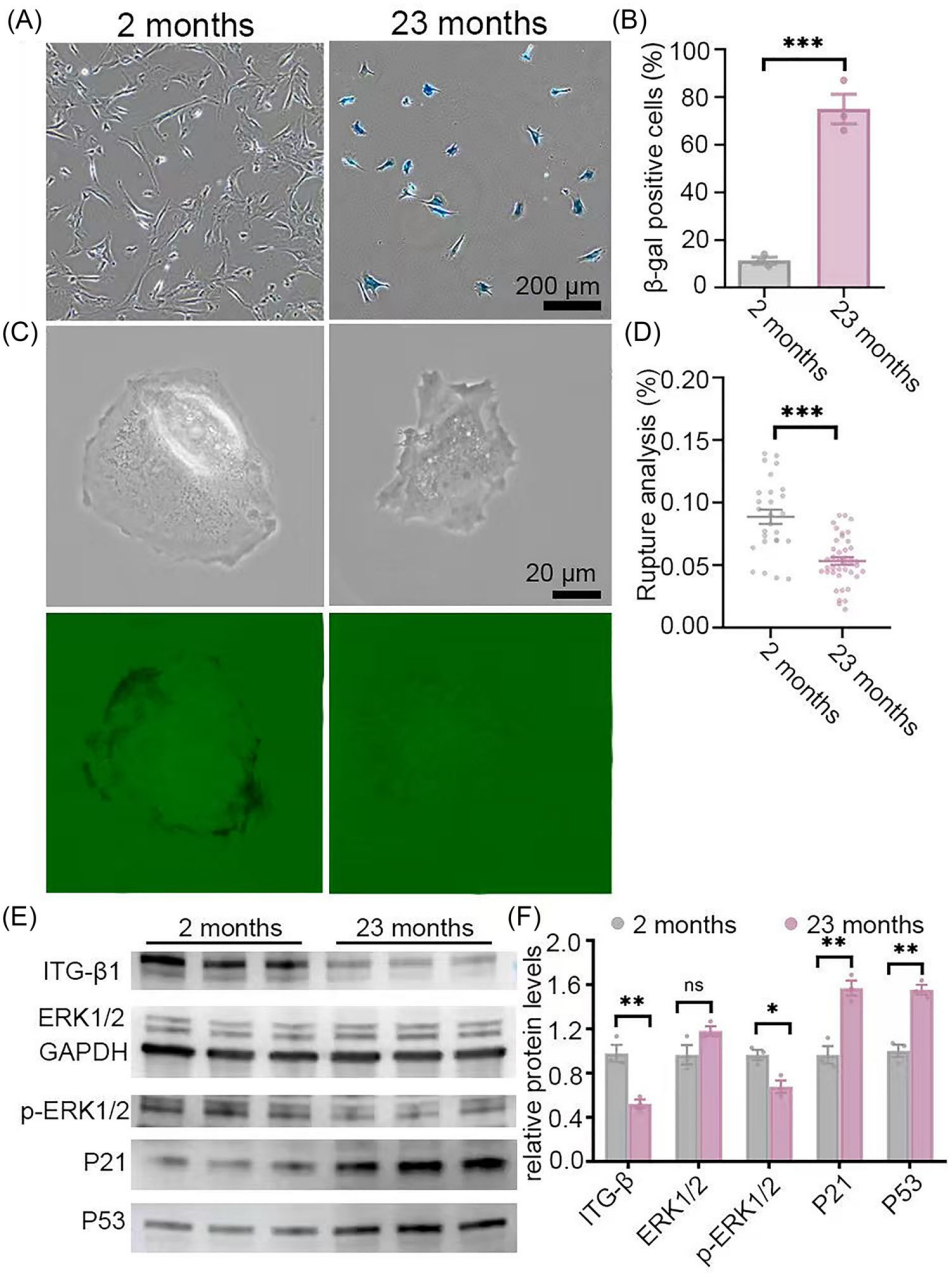
Adipose‐derived mesenchymal stem cells (AMSCs) from old and young mice were analyzed by the mechano‐probe. (A, B) β‐Gal staining of MSCs (A) along with the corresponding statistical analysis (B) demonstrating the aging status of the MSCs from young and old mice. (C, D) Force map of MSCs on the tension sensor surface (C) along with the corresponding statistical analysis (D). (E, F) Immunoblot analysis (E) along with the corresponding statistical analysis (F) showing the expression of ITG‐β1, ERK1/2, p‐ERK1/2, p21, and p53 in MSCs from young and old mice. Data are represented as mean ± SEM. Student's *t*‐test, *p < 0.05, **p < 0.01, ***p < 0.001, *n* = 3 in (A) and (B), *n* = 37 and 41 in (D).

After analyzing the aging status of MSCs by subculturing, we tried to confirm the efficacy of mechanical analysis of integrin tension of primary MSCs from animals. AMSCs were isolated from the adipose tissue of 2‐month‐old (young) and 23‐month‐old (aged) C57BL/6 mice. By the third passage, MSCs from aged (23‐month‐old) mice exhibited significantly slower proliferation and a markedly higher percentage of β‐galactosidase‐positive cells than those from young (2‐month‐old) mice (Figures 6A,B). Mechanical signals generated during GFP unfolding and/or avidin–biotin dissociation were lower in MSCs from aged mice compared to young MSCs (Figures 6C,D). Immunoblot analysis showed that aged MSCs had reduced p‐ERK and ITG‐β1 expression, accompanied by pronounced upregulation of p21 and p53, compared with young MSCs (Figures 6E,F). Together with integrin‐β1 tension profiles obtained from cell lines and primary MSCs (Figures 3, 4, 5, 6), these results provide strong support that GFP‐RGD mechano‐probes can reliably detect cellular senescence by monitoring adhesion‐mediated mechanical signaling.

However, in Figure 6, β‐gal staining demonstrated a pronounced shift in senescence status, with more than a sixfold increase, whereas the mechano‐probe readout decreased by approximately 40%–50% in AMSCs. This difference in dynamic range reflects the distinct biological parameters being measured. Integrin‐mediated tension operates within a relatively narrow functional window (typically >50 pN), constrained by cytoskeletal mechanics. Therefore, a 40%–50% reduction represents a substantial impairment in contractility that could significantly compromise cellular mechanical function and tissue integrity. In contrast, β‐gal enzymatic activity accumulates progressively and does not possess a defined physiological upper limit, allowing for large fold changes. Accordingly, the mechano‐probe inherently exhibits a narrower amplitude range than β‐gal staining. This distinction arises from measuring a bounded physiological parameter (force generation) versus an unbounded metabolic accumulation (enzymatic activity). Importantly, despite its relatively limited dynamic range, the mechano‐probe provides qualitative and functional information that high‐dynamic‐range senescence markers cannot offer. Moreover, the sensor enables analysis of live MSCs and allows cell reuse after measurement, representing a significant advantage over β‐gal staining, which requires fixation. Furthermore, variations in cell source and culture conditions may contribute to statistical variability. Therefore, mechano–probe‐based comparisons should be conducted under rigorously standardized and identical experimental conditions.

Our data here are based on RNAseq data, which showed a reduction in ITG‐β1 expression. However, other integrin subunits, such as ITG‐β2, ITG‐α3, ITG‐α6, and ITG‐β3 (Figures 1 and S6), were also detected as downregulated. The RGD‐recognizable integrin dimers include α5β1, α5β3, and αvβ3. In the RNAseq assay, α5 and αv were not detected, but they may also be downregulated. In future experiments, we should test whether rescuing other integrin subunits would result in a stronger contraction signal. This could also lead to the development of additional candidate ligands for use in mechanical probes. In addition, in our experiments here, MSCs were cultured in vitro on a 2D plastic surface and tested on a sensor‐coated glass surface. The passaging process and the non‐native environment following cell isolation could raise concerns about potential changes in the MSCs. Therefore, the development of in vivo mechano‐probes remains a critical goal for future research.

## 4 Conclusions

By comparing the mRNA profiles of young and aged MSCs, we developed a method for monitoring MSC aging through the analysis of effects on cell mechanics. We designed and used a novel biosensor for assessing the aging properties of MSCs by analyzing integrin tension. We designed a fluorescence protein‐based mechano‐probe to measure the cell mechanical property of integrin contraction. This probe monitors cell contractility by analyzing the interaction between ligand and cell membrane receptor, which advances the contraction test by means of gel contraction. The mechano‐probe does not encounter nuclease degradation of DNA‐based tension sensors, making our probe suitable for detecting integrin forces in MSCs, which contain nucleases in focal adhesions.

Using the mechano‐probe, we demonstrated that integrin tension per cell declines as MSCs age. This finding was supported by integrin β1 expression, and mechanistic studies suggest that the integrin β1/ERK signaling pathway contributes to reduced contractility. Conversely, upregulation of integrin β1 enhanced contraction in aged MSCs, indicating the possibility of altering aged MSCs by knocking‐in integrin to target cells. This approach also enables selecting out aged MSCs in a mixed cell pool of aged and young MSCs.

Although we have examined the decreased force signals by integrin expression, additional experiments are needed to understand the mechanism of reduced cell contraction in aged MSCs. Many kinases and gene expression pathways await further investigation.

## Author Contributions

**Yongliang Wang**: conceived the idea. **Yongliang Wang and Aimei Liu**: designed the research project. **Aimei Liu**: performed studies and data analysis. **Xiaojun Liu**: probe synthesis and data analysis. **Juan Li, Jiangtao Li, Xinpeng Wang, and Yuanjun Dong**: data analysis. **Aimei Liu and Yongliang Wang**: drafted the first version of the manuscript. **Xiaoyan Deng, Yongliang Wang, and Guixue Wang**: reviewed and edited the manuscript. All the authors read and approved the final manuscript.

## Conflicts of Interest

The authors declare no conflicts of interest.

## Supporting information




**Supporting Information**: nyas70247‐sup‐0001‐SuppMat.docx

## Data Availability

The data generated by this study are available in the article and its supporting information data files. All additional data supporting the findings of this study are available upon reasonable request by the corresponding author.
