# Supplementary material for: Detection of Mesenchymal Stem Cell Aging Using an Integrin Mechano‐Probe
Source: Ann N Y Acad Sci. 2026 Apr 9;1558:e70247. doi: 10.1111/nyas.70247 (PMC13064422; doi:10.1111/nyas.70247)
Supplement: Supplementary file 1 — Supporting Information: nyas70247‐sup‐0001‐SuppMat.docx [file NYAS-1558-0-s001.docx]

**Supplementary Materials**

**Title: Detection of mesenchymal stem cell aging using an integrin mechano-probe**

**Materials and methods**

**RT-qPCR**

Total RNA of cells was extracted using the TriZol Reagent (Invitrogen, Carlsbad, CA, USA) according to the manufacturer. cDNA was synthesized from total 1 μg RNA using the Reverse Transcription System (Promega) and 1 μl cDNA subjected to PCR using the Eco™ Real-Time PCR System (Illumina). Primers were listed in Supplementary Table 1. The expression level was evaluated using the 2^-ΔΔCt^ method.

**Cell proliferation assay**

**CCK-8 assay**: Cells (1 × 10^3^) were seeded in 96-well plates for 10 hours. Cell proliferation activity was measured using Cell Counting Kit-8 (CCK8, Dojindo Molecular Technologies, CK04-11) according to the manufacturer's protocol.

**Colony formation assay：**Cells (5×10^2^) were plated in six-well plates and grown for 10 days with the indicated treatment. Then, cells were fixed with 4% paraformaldehyde for 15 minutes and stained with 0.05% crystal violet for more than 1 hour. Colonies were counted with a dissecting microscope with which diameters larger than 200 μm were counted.

**Cell-Induced Senescence**

MSCs cells were treated with 200μM hydrogen peroxide for 24 hour followed by 2 days recovery, and stained with β-gal.

3T3-L1 Cells were treated with either (i) doxorubicin (50 nM, 48 hours) or (ii) hydrogen peroxide (200 μM, 24 hours). After treatment, cells were washed and cultured in normal medium for 2 days recovery, then stained for β-gal activity.

**Supplementary Table 1. Primers for RT-qPCR**

| **Genes** | **Forward Sequence** | **Reverse Sequence** |
| --- | --- | --- |
| ITG-β1 | GGATTCTCCAGAAGGTGGTTTCG | TGCCACCAAGTTTCCCATCTCC |
| P21 | AGGTGGACCTGGAGACTCTCAG | TCCTCTTGGAGAAGATCAGCCG |
| P53 | CCTCAGCATCTTATCCGAGTGG | TGGATGGTGGTACAGTCAGAGC |
| *GAPDH* | GTCTCCTCTGACTTCAACAGCG | ACCACCCTGTTGCTGTAGCCAA |

**Supplementary Fig. 1 RT-qPCR analysis of ITGB1, p53, and p21 mRNA expression in aged and young MSCs**


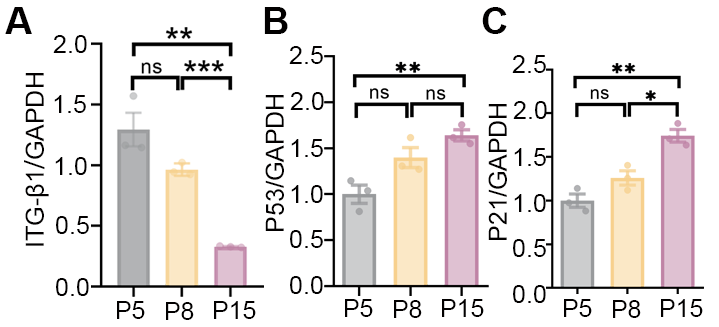


(A-C) The levels of ITG-β1**,** p53, and p21 mRNA were assessed by RT-qPCR. Data are represented as mean ± SEM, **P* < 0.05, ***P* < 0.01, ****P* < 0.001, One-way ANOVA analysis of variance with Tukey's multiple comparison, n = 3.

**Supplementary Fig. 2 The colony formation and proliferation ability of MSC at P5, P8 and P15**


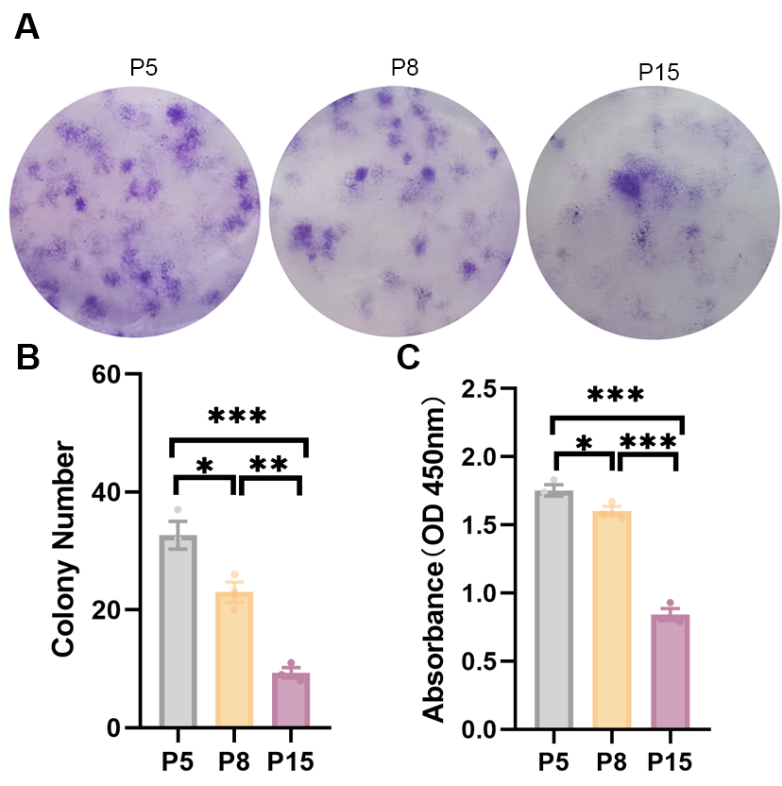


(A, B) The colony formation ability of MSC at P5, P8 and P15. (C) The proliferation ability of MSCS at P5, P8 and P15 was detected by CCK-8 assay. Data are represented as mean ± SEM. One-way ANOVA analysis of variance with Tukey's multiple comparison, n = 3. **P* < 0.05, ***P* < 0.01, ****P* < 0.001.

**Supplementary Fig. 3 The aging MSCs seeded on DNA probe surface**


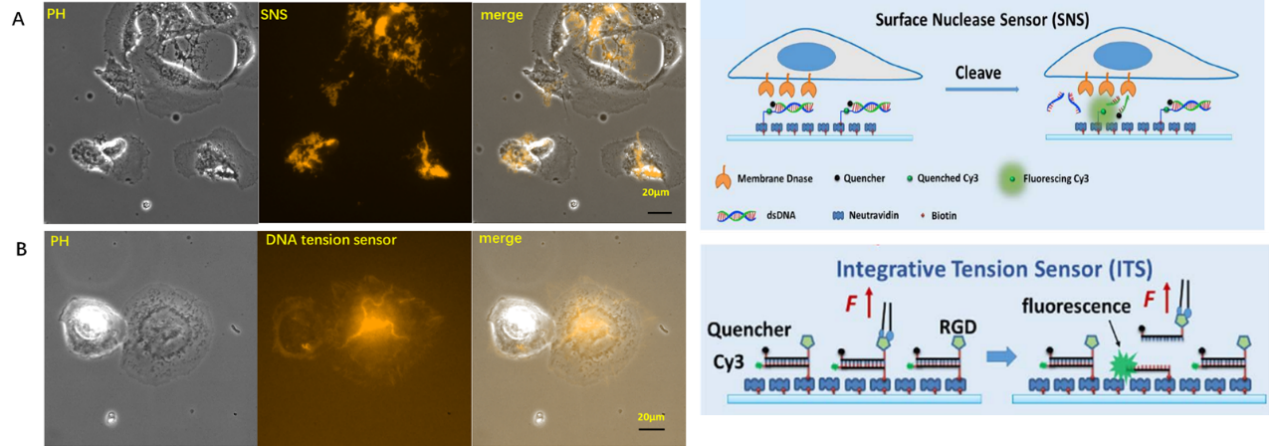


(A) the MSCs were seeded on surface nuclease sensors (J Biophotonics. 2019 Jan 28;12(5):e201800351.), the fluorescence is attributed to DNase signal. (B) the MSCs were seeded on 54pN ITS surface, the fluorescence is due to the DNase and integrin tension.

**Supplementary Fig. 4 The integrin tension was compared in 3T3-L1 at young and old states.**


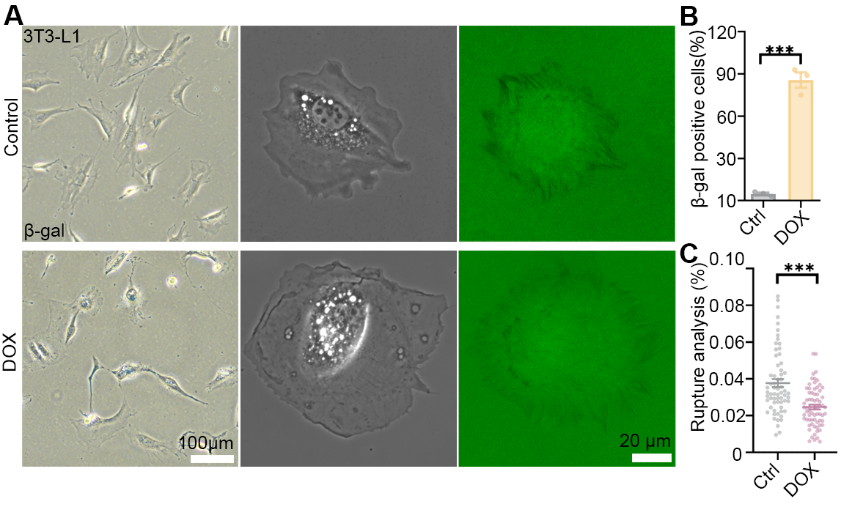


(A, B) DOX treatments induced 3T3-L1 senescence, as confirmed by β-gal staining in. (A, C) provided the force map of 3T3-L1 on the tension sensor surface along with the corresponding statistical analysis. Data are represented as mean ± SEM. Student's *t*-test. ****P*< 0.001. n=3 in B, n=60 in C.

**Supplementary Fig. 5 The H_2_O_2_-treated MSCs were analyzed by the mechano-probe**


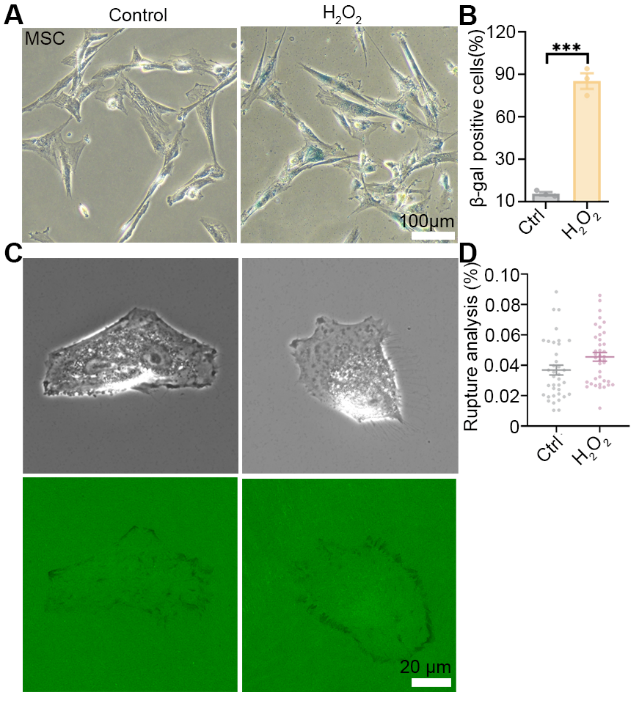


1. β-gal staining of H_2_O_2_-treated MSCs. (B) Comparison of β-gal positive MSCs between control (Ctrl) and H_2_O_2_-treated groups. (C) Force signal comparison reported by the mechano-probe in Ctrl and H_2_O_2_-treated MSCs. (D) Rupture analysis for both groups. Data are represented as mean ± SEM. Student's *t*-test. ****P*< 0.001. n=3 in B, n=30 in D.

**Supplementary Figure 6** **the mRNA level of integrin β3 in human MSCs**.


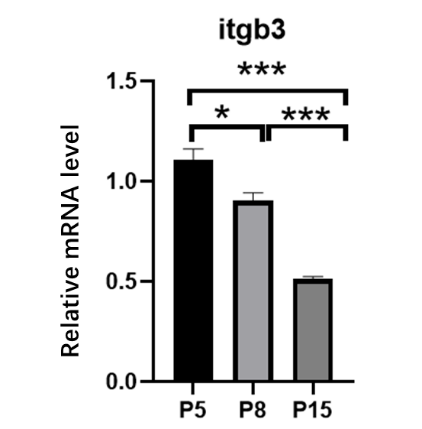


The levels of ITG-β3 mRNA were assessed by RT-qPCR. Data are represented as mean ± SEM, *P < 0.05, ***P < 0.001, One-way ANOVA analysis of variance with Tukey's multiple comparison, n = 3.
